# Review of the *Camponotus kiesenwetteri* group (Hymenoptera, Formicidae) in the Aegean with the description of a new species

**DOI:** 10.3897/zookeys.899.46933

**Published:** 2019-12-12

**Authors:** Sebastian Salata, Ana Carolina Loss, Celal Karaman, Kadri Kiran, Lech Borowiec

**Affiliations:** 1 Department of Entomology, California Academy of Sciences, San Francisco, CA, USA Department of Entomology, California Academy of Sciences San Francisco United States of America; 2 National Institute of Atlantic Forest (INMA), Santa Teresa, ES, Brazil National Institute of Atlantic Forest Santa Teresa Brazil; 3 Trakya University, Faculty of Sciences, Department of Biology, Balkan Campus, Edirne, Turkey Trakya University Edirne Turkey; 4 Department of Biodiversity and Evolutionary Taxonomy, University of Wrocław, Wrocław, Poland University of Wrocław Wrocław Poland

**Keywords:** Aegean Region, carpenter ants, *
Myrmentoma
*, new synonym, niche modelling, taxonomy

## Abstract

Based on recently collected material, the *Camponotus kiesenwetteri* group is redefined, and its members known from the Aegean region are diagnosed. *Camponotus schulzi***sp. nov.** is described from İzmir Province, Turkey. *Camponotus nadimi* Tohmé, 1969 **syn. nov.** is proposed as a junior synonym of *Camponotus libanicus* André, 1881 and *Camponotus kiesenwetteri cyprius* Emery, 1920 **syn. nov.** as a junior synonym of *Camponotus kiesenwetteri* (Roger, 1859). A key to workers of species of the *C. kiesenwetteri* group is provided. Niche modeling analyses are used to account for species habitat suitability across the Aegean region.

## Introduction

The genus *Camponotus* Mayr, 1861 with 1041 valid species and 454 valid subspecies is one of the most speciose within Formicidae. Members of this genus are distributed throughout the world, including the Arctic. However, unquestionably *Camponotus* reaches the highest diversity in the tropics (Bolton 2019). There are two regions in the Mediterranean (sensu Vigna Taglianti et al. 1999) that can be considered as centers of diversity of this genus. The first one, located on the western part of the Mediterranean, stretches from the Iberian Peninsula to the Atlas Mountains (Cagniant 1996; Fernandez 2019). The second one, located at the north-eastern edge of the Mediterranean, was defined by Fattorini (2000) as Aegean and covers the Balkans, western Turkey, Cyprus, Syria, Lebanon and northern Israel (Radchenko 1996; Tohmé and Tohmé 2000a, b; Ionescu-Hirsch 2010; Karaman 2012; Karaman and Aktaç 2013; Karaman et al. 2017; Salata and Borowiec 2018).

In the two last decades, the majority of studies on Mediterranean *Camponotus* focused on the Aegean region. Several recent publications show that this region is diverse and rich in taxa endemic to some islands (Borowiec and Salata 2014; Csősz et al. 2015; Salata and Borowiec 2015a, b, 2016, 2019; Salata et al. 2018) or mountain massifs (Csősz et al. 2007; Tinaut 2007; Kiran et al. 2008; Karaman and Aktaç 2013; Karaman et al. 2017; Salata and Borowiec 2017).

The *Camponotus kiesenwetteri* group comprises several taxa of the subgenus Myrmentoma Forel, 1912 distributed almost exclusively in the Aegean. Only *C. libanicus* André, 1881 and *C. aktaci* Karaman, 2013 extend their distribution range to Asia Minor and the Near East. For the first time, the group was defined by Emery (1925) as a group of taxa with impressed mesosomal dorsum, marginate propodeum, and matt body sculpture. Later Radchenko (1997) complemented the definition and listed the following species as members of the group: *C. aegaeus* Emery, 1915 *C. boghossiani* Forel, 1911, *C. kiesenwetteri* (Roger, 1859), and *C. libanicus*. However, the additional discoveries published in recent years provided a more comprehensive understanding of the diversity of the *kiesenwetteri* group (Karaman and Aktaç 2013, Salata and Borowiec 2018). Below, based on the material collected in the Aegean region, we update the definition of the *Camponotus kiesenwetteri* group, provide taxonomic diagnoses and distribution data for its known members and, based on material recently collected in Turkey, describe a new member of this group: *Camponotus schulzi* sp. nov. We also estimated habitat suitability in the Aegean region for species of the *C. kiesenwetteri* group.

## Material and methods

Specimens deposited in the Department of Biodiversity and Evolutionary Taxonomy, University of Wrocław, Poland and the Entomological Museum of Trakya University, Edirne, Turkey were collected between 1991 and 2019 from sites in different parts of the Aegean region. The dominant method was direct sampling (hand collecting). Individual specimens were collected on the ground and tree trunks and from low vegetation. Nests always were located in the soil, most often under trees. All specimens were preserved in 75% EtOH. The study was also supported by material deposited in the Natural History Museum of Crete (Iraklion, Greece), the Muséum d’Historie Naturelle, Genève, and samples collected by Petr Werner (Prague, Czechia). Photos were taken using a Nikon SMZ 1500 stereomicroscope, Nikon D5200 photo camera, and Helicon Focus software. All given label data are in the original spelling, presented in square brackets; a vertical bar (|) separates data on different rows and double vertical bars (||) separate labels. Type specimens’ photographs are available online on AntWeb (https://www.AntWeb.org) and are accessible using the unique CASENT or FOCOL identifying specimen code.

Examined specimens are housed in the following collections:

**DBET** Department of Biodiversity and Evolutionary Taxonomy, University of Wrocław, Poland;

**EMTU** Entomological Museum of Trakya University, Edirne, Turkey;

**MHNG**Muséum d’Historie Naturelle, Genève, Switzerland;

**MNHN**Muséum National d’Histoire Naturelle, Paris, France;

**MSNG**Natural History Museum, Genoa, Italy;

**NHMC**Natural History Museum of Crete, Iraklion;

**PW** Petr Werner collection, Prague, Czechia;

**ZMHB** Museum für Naturkunde der Humboldt-Universität, Berlin, Germany.

Pilosity inclination degree follows that used in Wilson (1955). Adpressed (0–5°) hairs run parallel or nearly parallel to the body surface. Decumbent hairs stand 10–40°, subdecumbent hair stands ~45° from the surface, suberect hairs bend about 10–20° from vertical, and erect hairs stand vertical or nearly vertical.

Measurements: all measurements are given in mm.

**HL** head length; measured in a straight line from mid-point of anterior clypeal margin to mid-point of posterior margin in full-face view;

**HW** head width; measured in full-face view directly above the eyes;

**SL** scape length; maximum straight-line length of scape;

**PW** pronotum width; maximum width of pronotum in dorsal view;

**PRL** propodeum length; measured in lateral view, from metanotal groove to posterior-most point of propodeum;

**PRW** propodeal width; maximum width of propodeum in dorsal view;

**PTH** petiole height; the chord of ventral petiolar profile at node level is the reference line perpendicular to which the maximum height of petiole is measured, measured in lateral view;

**PTW** petiole width; maximum width of the petiolar node in lateral view;

**WL** Weber’s length; measured as diagonal length from the anterior end of the neck shield to the posterior margin of the propodeal lobe.

Ratios:

**CI** cephalic index, HL/HW;

**SI** scape index, SL/HL;

**PI** petiole index, PTH/PTW.

Habitat suitability for species was estimated by niche modeling using Maxent 3.4.1 (Phillips et al. 2006) implemented in R package dismo (Hijmans et al. 2017). Niche modeling was estimated for all species with at least three distinct occurrence localities. The study region encompassed the Aegean biogeographic region as described by Fattorini (2000) with the addition of the Eastern Anatolian deciduous forest ecoregion, sensu World Wide Fund for Nature (WWF; Olson et al. 2001) (Fig. 36). As predictor variables we used solar radiation data and bioclimatic variables (derived from temperature and precipitation) from WordlClim version 2 (http://worldclim.org/version2) with 30 arc seconds spatial resolution grid. In order to minimize multicollinearity between variables, we ran a Pearson correlation analysis to identify variables with correlation absolute values equals or greater to 0.8. For each set of highly correlated variables, we kept only one variable, keeping the ones we consider more biologically meaningful for ant distribution. From an initial set of 31 variables, we selected 9: solar radiation of July (srad07), isothermality (bio03), temperature seasonality (bio04), maximum temperature of warmest month (bio05), minimum temperature of coldest month (bio06), mean temperature of wettest quarter (bio08), precipitation seasonality (bio15), precipitation of wettest quarter (bio16) and precipitation of warmest quarter (bio18). We used a 4-fold cross-validation test, with 75% of the data used for training and 25% for testing. For each species, all four replicates were averaged to build the final model. Importance of variables to the models were assessed by jackknife test. To avoid models that were no better than random, we only accepted final averaged models with a testing area under the curve (AUC) above 0.6.

### Synopsis of species of the *Camponotus kiesenwetteri* group

*Camponotus aegaeus* Emery, 1915

*Camponotus aktaci* Karaman, 2013

*Camponotus boghossiani* Forel, 1911

= *Camponotus boghossiani stenoticus* Emery, 1915 (= *Camponotus kiesenwetteri angustatus* Forel, 1889 not *Camponotus angustata* (Latreille, 1798))

*Camponotus kiesenwetteri* (Roger, 1859)

= *Camponotus kiesenwetteri cyprius* Emery, 1920 **syn. nov.**

*Camponotus libanicus* André, 1881

= *Camponotus libanicus sahlbergi* Forel, 1913

= *Camponotus nadimi* Tohmé, 1969 **syn. nov.**

*Camponotus nitidescens* Forel, 1889

*Camponotus schulzi* **sp. nov.**

## Taxonomy

### Camponotus kiesenwetteri

Taxon classificationAnimaliaHymenopteraFormicidae

group

F7F4A6C8-5989-53BE-96F0-03BCB6197792

#### Diagnosis.

Metanotal groove absent or shallow; propodeal dorsum relatively flat, propodeal declivity deeply concave, posterior protrusions absent or weakly to well developed; body densely punctate, appears dull (only *C. nitidescens* and *C. schulzi* have sculpture partially reduced on the lateral sides of mesosoma); the whole body bearing short to long, thick, pale and erect setae, and additional short appressed microsetae; head, mesosoma, and gaster uniformly blackish-brown to black (only *C. aktaci* has gaster yellowish-brown); polymorphic species.

#### Biology.

All known species have similar biological preferences and were most often collected in warm and arid habitats within coniferous forests, especially pine forests. Less frequently they were observed in oak forest, woodland-meadow ecotones, xerothermic meadows, suburban areas with maquis, pastures with shrubs, olive plantations, river bank, orchards, occasionally in rocky gorges with deciduous trees. However, records from open habitats most often were located in the vicinity of trees, especially pine trees. Nests were located in soil, usually sandy, under trees, most often between roots, under small stones, less frequently under big stones. The only observed nest of *C. nitidescens* was located in a cracked rock wall on a roadside in oak forest under a loose piece of rock. Workers were active all day with the highest activity at dusk. Both major and minor workers were most often found on trunks and branches of coniferous trees, less often on the ground or litter.

Most of the records located in the European mainland came from areas below 700 m a.s.l. and only *C. nitidescens* is known exclusively from sites located between 1100 and 1700 m a.s.l. However, on Crete, specimens of *C. kiesenwetteri* were also found in area above 1000 m a.s.l., and the highest record comes from Trocharis peak in Lasithi province (2131 m a.s.l.). Members of the group known from Turkey manifest more alpine preferences. According to label data, the new species *Camponotus schulzi* was collected at the site located at an altitude of 1150–1500 m. Also *C. aktaci* is known almost exclusively from montane habitats located above 1000 m a.s.l.

## 

### A key to workers of species of the *Camponotus kiesenwetteri* group

**Table:** 

1	Mesosoma in lateral view forms a regular arch; metanotal groove absent (Figs 17–22)	**2**
–	Mesosoma in lateral view with shallow metanotal groove (Figs 2, 6, 11–16)	**4**
2	Legs mostly yellowish to reddish-brown, gaster yellowish-brown. Setation of head, mesosoma, and gaster short and sparse (Figs 21, 22). Eastern, western and central Turkey (Fig. 25)	***C. aktaci* Karaman**
–	Legs and gaster mostly brown to black. Setation of head, mesosoma, and gaster long and dense (Figs 17–20)	**3**
3	Petiolar scale thin, PI > 1.50 (Figs 17, 18). Northeastern Greece, Eastern Aegean Islands and western Turkey (Fig. 24)	***C. aegaeus* Emery**
–	Petiolar scale thick, PI < 1.42 (Figs 19, 20). The Middle East (Fig. 32)	***C. libanicus* André**
4	Posterior margin of propodeum with well developed, lateral dentate protrusions (Figs 13, 14). Base of antennal scape with extension. Northeastern, eastern and southern Greece and western Turkey (Fig. 30)	***C. kiesenwetteri* (Roger)**
–	Posterior margin of propodeum without or with weakly developed, indistinct protrusions (Figs 2, 11, 12, 15, 16). Base of antennal scape without or with indistinct extension	**5**
5	Surface of mesosoma more strongly sculptured, reticulate and granulate with more or less dull background; posterior margin of propodeum sometimes with weakly-developed, indistinct protrusion (Figs 11, 12). Base of antennal scape without extension. Peloponnese, Crete, southern and eastern Aegean islands and western Turkey (Fig. 28)	***C. boghossiani* Forel**
–	Surface of mesosoma weaker sculptured, especially sides of mesosoma appear more or less shiny; posterior margin of propodeum without protrusions (Figs 1, 2, 5, 6, 15, 16). Base of antennal scape with or without extension	**6**
6	Base of antennal scape with extension (Fig. 3). Petiolar scale thick, PI: 1.26–1.33 (Figs 1, 2, 5, 6). Western Turkey (Fig. 35)	***C. schulzi* sp. nov.**
–	Base of antennal scape without extension (Fig. 4). Petiolar scale thin, PI: 1.54–1.74 (Figs 15, 16). Cephalonia Island, western Sterea Ellas and Peloponnese (Fig. 34)	***C. nitidescens* Forel**

### Camponotus aegaeus

Taxon classificationAnimaliaHymenopteraFormicidae

Emery, 1915

0FFEBFB3-EE73-5BA5-9397-F450D98E76FB

Figs 17 , 18 , 23 , 24

Camponotus (Orthonotomyrmex) libanicus var. aegaea Emery, 1915: 4, figs 1, 2 (s.w.q.m.). Syntype workers, queen, Isola Rodi, Greece (Festa) (MSNG) [Syntype worker images examined, AntWeb, CASENT0905395, photos by Zach Lieberman, available on https://www.AntWeb.org]

#### Diagnosis.

Head, mesosoma, and gaster uniformly blackish-brown to black; metanotal groove absent; propodeum without posterior protrusion; body densely punctate, appears dull; base of scape without extension; whole body bears long, thick, pale, dense and erect setae, and short appressed microsetae; petiolar scale thin (PI > 1.50).

#### Distribution.

Greece: North Aegean Islands, South Aegean Islands (Dodecanese), Central Macedonia, Eastern Macedonia and Thrace; Turkey: Adana, Afyon, Antalya, Aydın, Balıkesir, Bilecik, Bursa, Çanakkale, Denizli, Diyarbakır, Elazığ, İzmir, Kırklareli, Kütahya, Manisa, Muğla, Sakarya, Uşak, and Yalova. The species was also recorded from North Macedonia (Bračko et al. 2014) and Bulgaria (Lapeva-Gjonova 2010).

#### Comments.

Almost completely blackish-brown to black body and regularly arched (in lateral view) mesosoma cluster this species with *Camponotus libanicus*. At first glance both species look extremely similar and the most relevant character distinguishing both taxa is the shape of petiolar scale. *Camponotus aegaeus* has the scale thin (PI > 1.50) with a feebly convex anterior surface, while in *C. libanicus* the scale is thick (PI < 1.42) with a strongly convex anterior surface. Both species appear to be vicariant taxa with a more westerly distribution of *C. aegaeus* and more a easterly distribution of *C. libanicus* (Figs 24, 32). Indeed, niche modeling for both species show similar areas with high suitability, especially along the south coast of Turkey and Cyprus. However, unlike *C. libanicus*, *C. aegaeus* has not been recorded from the island. Solar radiation was the variable that contributed the most to the niche model of *C. aegaeus*.

### Camponotus aktaci

Taxon classificationAnimaliaHymenopteraFormicidae

Karaman, 2013

B0A3AB8B-CC28-5A6D-B860-DA033DD68B12

Figs 21 , 22 , 25 , 26

Camponotus aktaci Karaman, 2013: 37, figs 1, 7 (w.). Holotype worker, Akcatekir Village, (37°21’N, 34°49’E), 1300 m a.s.l., Adana, Turkey (EMTU) [holotype and paratypes personally investigated].

#### Diagnosis.

Head and mesosoma uniformly black, gaster and legs yellowish-brown; metanotal groove absent; propodeum without posterior protrusion; body densely punctate, appears dull; base of scape without extension; whole body bears short, thin, pale, sparse and erect setae, and short appressed microsetae; petiolar scale thick.

#### Distribution.

Turkey: Adana, Bingöl, Diyarbakır, Elazığ, Malatya, Muğla.

#### Comments.

Mostly yellowish-brown gaster and legs and short and sparse setation of head, mesosoma and gaster distinctly separates this species from other members of the *Camponotus kiesenwetteri* group. Temperature seasonality contributed most to the distribution model. Niche modeling showed highly suitable areas matching species known distribution at Eastern Anatolian deciduous forests but also additional areas in the central Anatolian steppe region, where there are no current occurrence records for the species. However, the westernmost record from Muğla Province is located in an area of low habitat suitability.

### Camponotus boghossiani

Taxon classificationAnimaliaHymenopteraFormicidae

Forel, 1911

732F6E79-9BE8-5791-8498-1EE6E1AAF7BF

Figs 11 , 12 , 27 , 28

Camponotus boghossiani Forel, 1911: 357 (s.w.). Syntype workers, Lesbos, Greece (MHNG) [syntypes personally investigated, CASENT0910435 and CASENT0910436].
=Camponotus boghossiani var. stenotica Emery, 1915: 7 (=Camponotus kiesenwetteri angustatus Forel, 1889: 261, not Camponotus angustata (Latreille, 1798)); Salata and Borowiec 2018: 7: as a synonym of C. boghossiani. Holotype worker, Samos, Greece (ZMHB) [Holotype worker images examined, AntWeb, FOCOL2488, photos by Christiana Klingenberg, available on AntWeb.org]. Note: specimen from Rethymno, Crete, Greece (MSNG), CASENT0905396 is wrongly noted as syntype of Camponotus stenoticus.

#### Diagnosis.

Head, mesosoma, and gaster uniformly black; metanotal groove present, shallow; propodeum without or with indistinct bulge-like protrusions; body densely punctate, appears dull; base of scape without extension; whole body bears long, thick, pale, dense and erect setae, and short appressed microsetae; petiolar scale thick.

#### Distribution.

Greece: North Aegean Islands, Crete (Heraklion), South Aegean Islands (Cyclades, Dodecanese), Peloponnese (Messinia); Turkey: Antalya, Balıkesir, Çanakkale, Denizli, Karaman, Kütahya, Muğla, and Uşak.

#### Comments.

Density of sculpture slightly differs within this species and populations from Peloponnese and Aegean Islands are slightly more sculptured than populations from western Turkey. *Camponotus boghossiani* is most similar to *C. nitidescens* and *C. schulzi* and differs from them in the stronger sculpture of the mesosoma and gaster which, at first glance, appears very dull. While in both relatives the sculpture is slightly diffused and the surface is at least partly shiny. *Camponotus kiesenwetteri* has a similarly sculptured body surface but differs in having the posterior margin of the propodeum more or less excavate and forming well-developed, lateral dentate protrusions while in *C. boghossiani* the posterior margin of the propodeum is straight, without protrusions. Isolated specimens of *C. kiesenwetteri*, with posterior margin of propodeum very shallowly excavate, at first glance look very similar to specimens of *C. boghossiani* but can be easily be separated by having an antennal scape with a distinct basal extension, while in *C. boghossiani* the base of the antennal scape has no extension. Precipitation of the wettest quarter was the variable that contributed the most to the distribution model. High suitable areas are indicated especially along the coast of Turkey, Cyprus and Crete.

### Camponotus kiesenwetteri

Taxon classificationAnimaliaHymenopteraFormicidae

(Roger, 1859)

1A30538B-FCCB-5BEF-A4AB-1AB43042DC15

Figs 13 , 14 , 29 , 30

Formica (Hypoclinea) kiesenwetteri Roger, 1859: 241 (w.). Syntype workers, Greece (ZMHB) [Syntype workers images of Formica (Hypoclinea) kiesenwetteri examined, AntWeb, FOCOL2486 and FOCOL2487, photos by Christiana Klingenberg, available on https://www.AntWeb.org].
=Camponotus kiesenwetteri var. cypria Emery, 1920: 26 (w.) **syn. nov.** Syntype worker, Cyprus (MSNG) [Syntype worker images of Camponotus kiesenwetteri cyprius examined, AntWeb, CASENT0905397, photos by Zach Lieberman, available on https://www.AntWeb.org]

#### Diagnosis.

Head, mesosoma, and gaster uniformly black; metanotal groove present, shallow; propodeum with distinct dentate protrusions; body densely punctate, appears dull; base of scape with extension; whole body bears long, thick, pale, dense and erect setae, and short appressed microsetae; petiolar scale thick.

#### Distribution.

Greece: Attica, North Aegean Islands, South Aegean Islands (Cyclades, Dodecanese), Central Greece, Crete (Chania, Heraklion, Lasithi, Rethymno), Ionian Islands, Central Macedonia, Eastern Macedonia and Thrace, Peloponnese; Cyprus; Turkey: Balıkesir, İzmir and Muğla.

#### Comments.

The species can be easily separated by the following combination of characters: strongly sculptured body, mesosoma with metanotal groove and posterior margin of propodeum with distinct dentate protrusions, and antennal scape with distinct basal extension. *Camponotus nitidescens* and *C. schulzi* both differ in having a partly shiny body, and *C. boghossiani* differs in having a propodeum without apical protrusions and an antennal scape without basal extension.

*Camponotus kiesenwetteri cyprius* was described by Emery (1920) based on four specimens collected from Cyprus (no data indicating a precise location). The subspecies was separated from the typical form based on the following characters: smaller body, wider mesosoma, indistinct metanotal groove, thicker petiole and shape of propodeal protrusions. The investigated type specimen agrees with the mentioned description but some of those characters overlap with intraspecific variability observed within *Camponotus kiesenwetteri*. Thus, we consider this species a junior synonym of *C. kiesenwetteri*. Nonetheless, Cyprus did not appear as a suitable region in niche modelling. Minimum temperature of coldest month was the variable that contributed most to the distribution model.

### Camponotus libanicus

Taxon classificationAnimaliaHymenopteraFormicidae

André, 1881

EBFDD4DC-149E-51FA-A00F-2ABB6EA824B7

Figs 19 , 20 , 31 , 32

Camponotus (Orthonotomyrmex) libanicus André, 1881: 54, pl. 3, figs 14, 15 (w.). Syntype worker, Lebanon (MNHN) [Syntype worker images examined, AntWeb, CASENT0913700, photos by Will Ericson, available on https://www.AntWeb.org].
=Camponotus (Orthonotomyrmex) libanicus r. sahlbergi Forel, 1913: 435 (s.w.); Radchenko 1996: 1197, as a synonym of C. libanicus. Syntype worker, Bolkar Mountains, Turkey (MHNG) [Syntype workers images examined, AntWeb, CASENT0910441, and CASENT0910440, photos by Zach Lieberman, available on https://www.AntWeb.org].
=Camponotus (Myrmentoma) nadimi Tohmé, 1969: 6, figs 3, 4 (s.w.) **syn. nov.** [types unavailable].

#### Diagnosis.

Head, mesosoma, and gaster uniformly black; metanotal groove absent; propodeum without posterior protrusion; body densely punctate, appears dull; base of scape without extension; whole body bears long, thick, pale, dense and erect setae, and short appressed microsetae; petiolar scale thick (PI < 1.42).

#### Distribution.

The species is known from Lebanon (André 1881, Tohmé 1969) and Cyprus: Limassol and Girne. It was also recorded from Adana, Diyarbakır, Elazığ, Hatay, Karaman, and Mersin provinces in Turkey (Forel 1913; Emery 1915; Bolu and Özgen 2018), Israel (Ionescu-Hirsch 2010) and Iran (Paknia et al. 2010). Record from Greece: Aegean Islands by Legakis (2011) is based on unpublished manuscript (Taylor and Clee 2008) and is likely based on a misidentification. Recent research on the ant fauna of the Aegean Islands has not confirmed the occurrence of this species in Greece. Additionally, the old record from İzmir in Turkey (Forel 1911) is doubtful as it was published before the description of *C. aegaeus* and it is located 500 km West of all the recently known localities of this species.

#### Comments.

*Camponotus libanicus* belongs to the species with mesosoma evenly convex in profile, not interrupted by the metanotal groove. It is very similar to *C. aegaeus* and differs by having a thick petiolar scale with PI < 1.42, which in *C. aegaeus* is thinner at PI > 1.50. See also comments in *C. aegaeaus*.

In the description of *C. nadimi* from Lebanon, Tohmé (1969) compared this species with *C. libanicus*. The author noted that *C. nadimi* is distinctly polymorphic, while *C. libanicus* was considered as almost monomorphic. Additionally, *C. nadimi* was differentiated from *C. libanicus* based on the presence of emargination on the anterior margin of the clypeus and a thinner petiole. Ionescu-Hirsch (2010) was the first to suggest that the characters mentioned in the description overlap with intraspecific variability observed within populations of *C. libanicus*. Our observations confirm this and, additionally, samples investigated during our study consisted of distinctly polymorphic specimens. Therefore, we consider *C. nadimi* a junior synonym of *C. libanicus*. Minimum temperature of the coldest month was the variable that contributed most to the distribution model. Highly suitable areas are indicated specially along the coast of Turkey, Cyprus, Crete and Eastern Mediterranean conifer forests.

### Camponotus nitidescens

Taxon classificationAnimaliaHymenopteraFormicidae

Forel, 1889

A80E55CB-6C8E-5CD9-8D1F-E3BB5DBE5581

Figs 4 , 15 , 16 , 33 , 34

Camponotus kiesenwetteri nitidescens Forel, 1889: 260 (w.) Syntype workers, Kefalonia, Greece (MHNG) [syntypes personally investigated, CASENT0910437 and CASENT0910438].

#### Diagnosis.

Head, mesosoma, and gaster uniformly brownish-black to black; metanotal groove present, shallow; propodeum without protrusions; body punctate, mesosoma with sculpture reduced and its lateral sides at least partially shiny; base of scape without extension; whole body bears long, thick, pale, dense and erect setae, and short appressed microsetae; petiolar scale thick.

#### Distribution.

Greece: Ionian Islands (Cephalonia) Peloponnese (Lakonia and Messinia), Western Greece (Aetolia-Acarnania).

#### Comments.

*Camponotus nitidescens* together with *C. schulzi* are well distinguished from other species of the *C. kiesenwetteri* group in the partly reduced sculpture of the mesosoma and gaster with, at least, the lateral sides of mesosoma partly shiny. However, the sculpture is never as shiny as in members of related members of the *Camponotus lateralis* group. Solar radiation was the variable that contributed the most to the distribution model. Although the known distribution is restricted to the western area of the Aegean region, highly suitable areas are indicated in Crete, northeast coast of Turkey, coast of Syria and Lebanon.

### Camponotus schulzi sp. nov.

Taxon classificationAnimaliaHymenopteraFormicidae

27B5E1DB-6172-5E27-B498-B768DFD8B8D3

http://zoobank.org/A9B66F54-26A8-44BE-BD39-4A0BEC973F8E

Figs 1–4 , 5, 6 , 7, 8 , 9–10 , 35

#### Type material.

***Holotype***: major worker (CASENT0876000): Turkey |Bozdag Mountain | 38.3277N, 28.1112E || 1150–1500 mH | 10.05.2003 | leg. A. Schulz (DBET); ***paratypes***: 2 major workers, 5 minor workers (CASENT0876001–CASENT0876007): the same data as holotype (DBET, PW, EMTU).

#### Diagnosis.

Head, mesosoma, and gaster uniformly black; metanotal groove present, shallow; propodeum without protrusions; body punctate, mesosoma with sculpture reduced and its lateral sides at least partially shiny; base of scape with extension; whole body bears long, thick, pale, dense and erect setae, and short appressed microsetae; petiolar scale thick.

#### Description.

**Figures 1–4.**
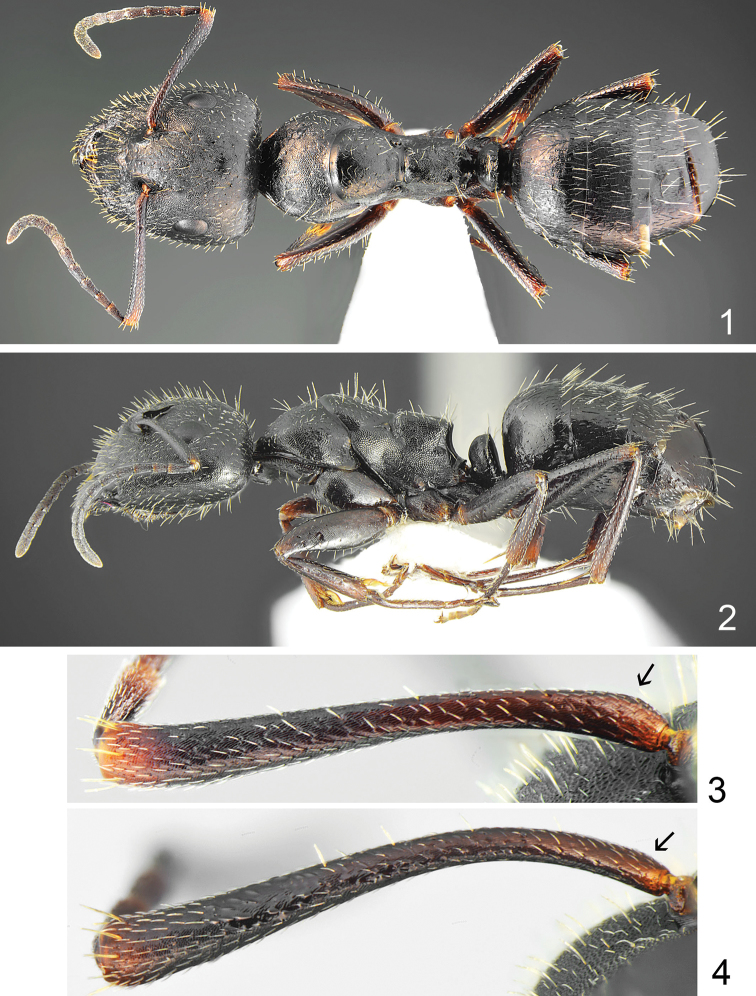
**1, 2***Camponotus schulzi* sp. nov., major worker: **1** dorsal **2** lateral **3, 4** antennal scape **3***Camponotus schulzi* sp. nov. **4***Camponotus nitidescens* (arrows indicate the base of scape lacking extension).

**Figures 5, 6.**
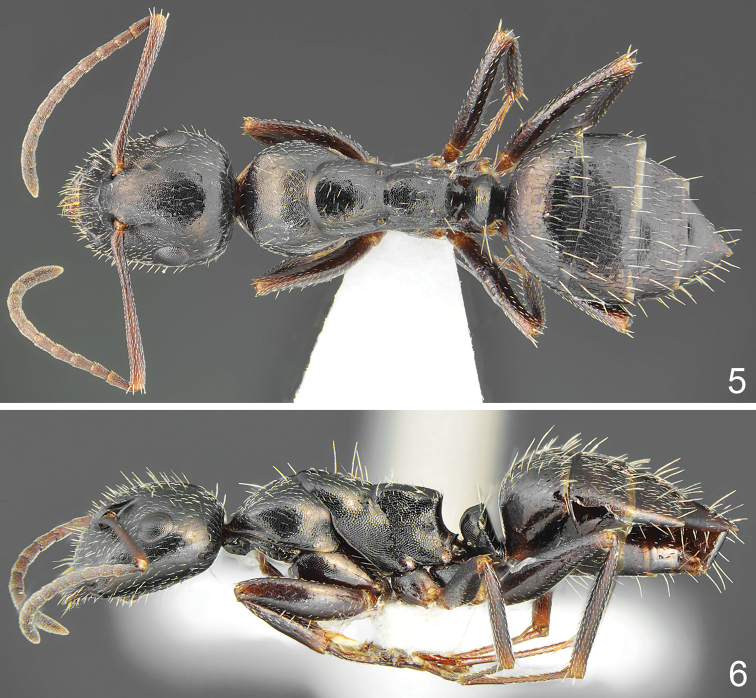
*Camponotus schulzi* sp. nov., minor worker **5** dorsal **6** lateral.

**Figures 7, 8.**
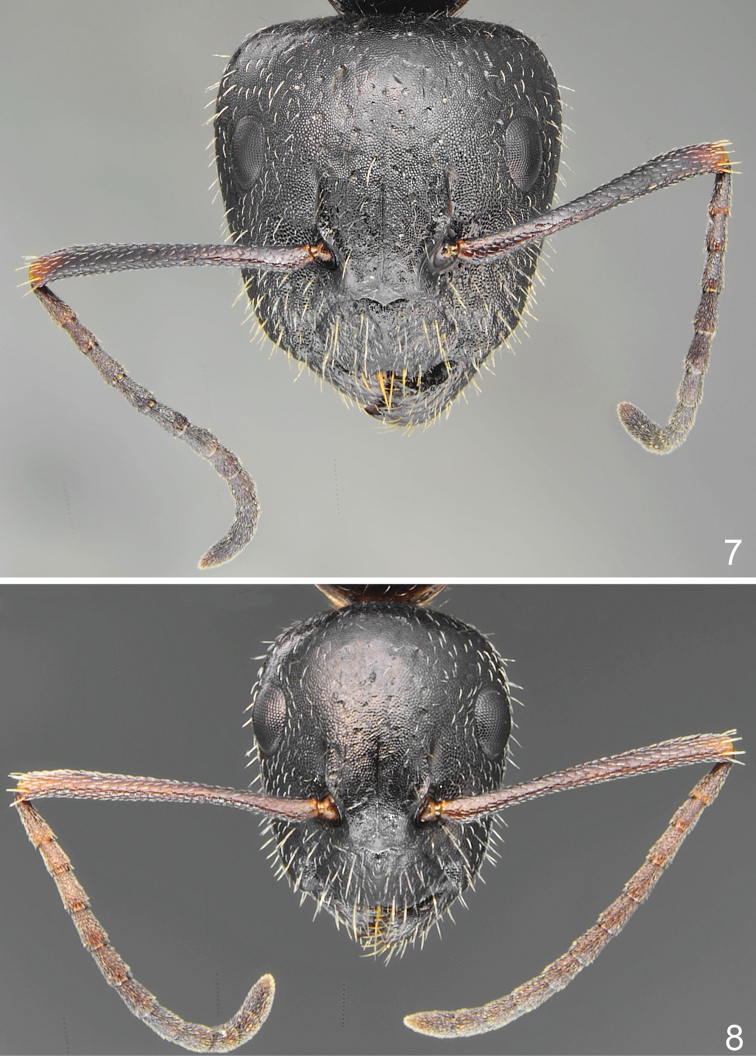
*Camponotus schulzi* sp. nov., head and antennae **7** major worker **8** minor worker.

***Measurements*.** Major worker (n = 3): HL: 1.827 (1.78–1.92), HW: 1.72 (1.63–1.82), SL: 1.59 (1.52–1.65), WL: 2.343 (2.27–2.44), PW: 1.22 (1.16–1.27), PRL: 0.657 (0.64–0.68), PRW: 0.43 (0.42–0.44), PTH: 0.40 (0.38–0.41), PTW: 0.293 (0.27–0.32), CI: 1.041 (1.028–1.055), SL/HW: 0.926 (0.889–0.982), PTH/PTW: 1.367 (1.281–1.413); minor worker (n = 5): HL: 1.31 (1.13–1.46), HW: 1.03 (0.94–1.29), SL: 1.297 (1.21–1.41), WL: 1.83 (1.65–2.02), PW: 0.96 (0.86–1.08), PRL: 0.58 (0.52–0.64), PRW: 0.34 (0.32–0.39), PTH: 0.397 (0.35–0.48), PTW: 0.307 (0.27–0.38), CI: 1.192 (1.132–1.241), SI: 1.185 (1.093–1.287), PI: 1.297 (1.263–1.333). ***Body colouration.*** Head, mesosoma and petiolus black, gaster from brownish-black to black. Legs brown to black, trochanters as dark as femora (Figs 1, 2, 4, 5), antennal scape brown, base and apex of scape in some specimens paler than the central part of scape, reddish-brown (Fig. 3). ***Head.*** In major workers large, trapezoidal in outline, the widest at height of eyes, distinctly narrowed anteriorly and rounded posteriorly (Fig. 7). Anterior margin of clypeus in the middle with semicircular emargination. Eyes small, placed distinctly below the mid-length of the head, 0.6 times as long as the length of tempora and 0.47 times as long as the length of genae. Scape short, slightly shorter than the width of head, with well-marked extension, without preapical constriction (Fig. 3). Funicle elongate and thin, 1.3 times as long as scape, first segment elongate, 2.3–2.4 times as long as wide on the apex, 1.4 times as long as the second segment, segments 3–6 equal in length and slightly longer than the second segment, segments 7–11 slightly shorter than the second segment. Surface of scape with fine microsculpture, very short and sparse appressed setae and 2–3 short, erect setae (Fig. 7). In minor workers head oval, the widest at height of eyes; slightly narrowed anteriorly and rounded posteriorly (Fig. 8). Anterior margin of clypeus without or with very shallow emargination. Eyes proportionally larger than in major workers; placed distinctly below the mid-length of the head, small, approximately 0.78 times as long as the length of tempora and 0.56 times as long as the length of genae. Scape short, slimmer than in major workers, 1.2–1.3 times longer than the width of head, with well-marked extension, without preapical constriction. Funicle in shape and ratio of segments similar to major workers. The surface of scape with fine microsculpture, covered with very short and sparse appressed setae, without erect setae. The whole surface of the head, in both major and minor workers, with numerous white, erect setae (Figs 2, 6). Mandibles short, dorsal surface with distinct microreticulation and partly with elongate setose punctures and elongate rugulae, matt, inner margin with one larger and 3–4 smaller teeth. Clypeus on the whole surface microreticulate and with sparse, moderately coarse, setose punctures, matt. Frontal carinae short, extending to the line connecting 1/3 length of eyes, form a regular arch, antennal sockets flat with a thin median line, microreticulate, with sparse setose punctures, dull. The area between eyes and occipital margin of head distinctly microreticulate and appears distinctly dull, microreticulation gradually diffused from dorsal to the ventral part of the head. Gena and tempora on the underside of the head with interspaces microreticulate to granulate, shiny. ***Mesosoma.*** Promesonotum regularly convex in profile with distinct metanotal groove, slightly deeper in major workers than in minor workers (Figs 2, 6). Propodeum elongate, in major workers 1.36–1.40 and in minor worker 1.50–1.60 times as long as wide; dorsal surface flat, posterior margin distinctly concave, posterior corners never forming tooth-like protrusions. The whole surface of pronotum, dorsal part of mesonotum and lateral parts of propodeum with sparse, moderately long, appressed setae, dorsal part of the whole mesosoma with long, white erect setae. Mesosoma on dorsal surface with distinct microreticulation, cells of microsculpture with shiny interspaces. On lateral sides of pronotum, microreticulation tending to form a linear sculpture of slightly shiny interspaces, sides of meso- and metathorax with a regular granulate sculpture of slightly shiny to matt interspaces. ***Petiole.*** Microreticulate but appears shiny. Petiolar squama stout, 1.26–1.33 as high as wide in lateral view, with convex anterior and flat posterior surfaces, margin with row of long, white setae (Figs 2, 6). ***Gaster.*** Tergites with sparse, short appressed setae and numerous long erect setae, with distinct regular microsculpture of transverse cells, on the whole surface more or less shiny. ***Legs.*** Moderately long, hind femora 0.8 times as long as mesosoma, hind tibiae slightly shorter than hind femora, the first segment of hind tarsi 0.8 times as long as hind femora. The whole surface of femora and tibiae with short, sparse, appressed to suberect pubescence, posterior and ventral surface of fore femora, and ventral surface of mid and hind femora with several, long erect setae, the surface of femora and tibiae appear shiny to slightly matt. Hind tibia with one long and two short apical spines and on the inner surface with a row of 3–5 short spines.

#### Etymology.

Named after Andreas Schulz, a German amateur myrmecologist and naturalist, who extensively explored the Aegean region and collected valuable material, including the specimens of *C. schulzi* sp. nov.

#### Distribution.

Western Turkey: İzmir Province, Bozdağ Mts.

**Figures 9, 10.**
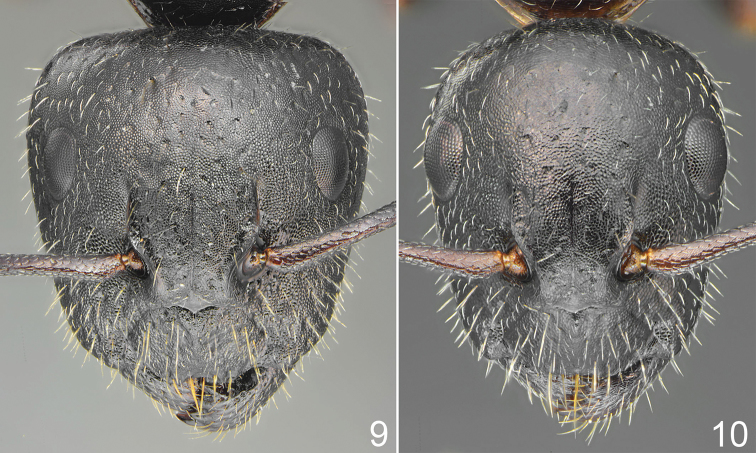
*Camponotus schulzi* sp. nov., head sculpture **9** major worker **10** minor worker.

#### Comments.

**Figures 11–16.**
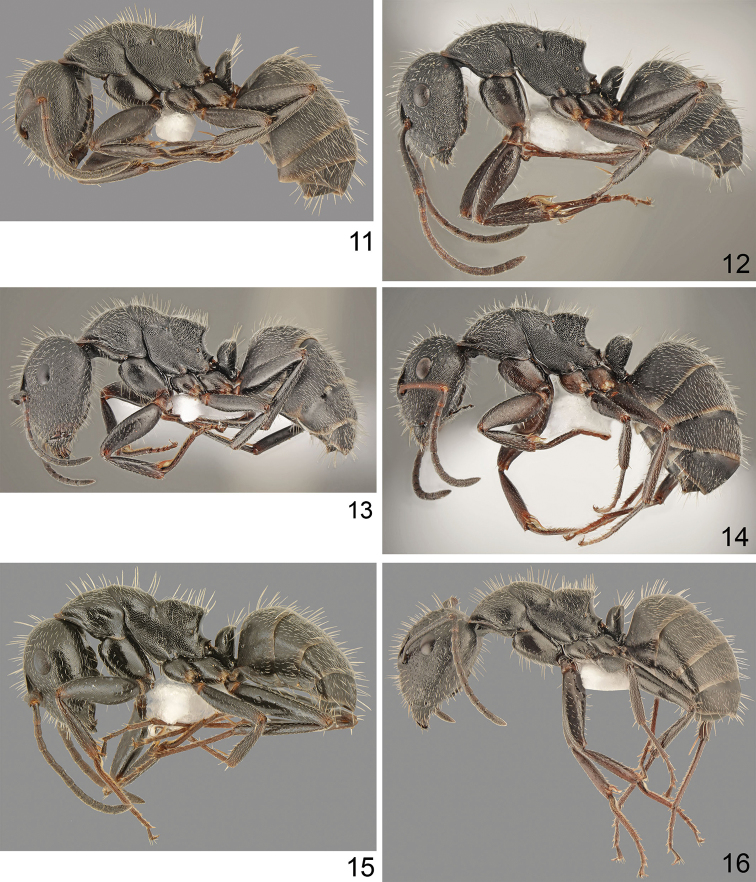
Workers in lateral view **11, 13, 15** major **12, 14, 16** minor: **11, 12***Camponotus boghossiani* Forel **13, 14***C. kiesenwetteri* (Roger) **15, 16***C. nitidescens* Forel.

*Camponotus schulzi* sp. nov. is distinctly polymorphic, the largest major workers 1.5 times longer than the smallest minor workers. Within the *C. kiesenwetteri* group, together with *C. boghossiani*, *C. kiesenwetteri*, and *C. nitidescens*, it forms a distinct complex characterized by a shallow but distinct metanotal groove. *Camponotus boghossiani* and *C. kiesenwetteri* differ from *C. schulzi* in the matt body with strong and non-reduced sculpture on the whole head, mesosoma, and gaster (Figs 11–14). Additionally, *C. kiesenwetteri* differs in having well-developed, dentate protrusions on the posterior margin of propodeum, while in *C. schulzi* sp. nov. the posterior margin of the propodeum is lacking such structures; *C. boghossiani* differs also in the base of antennal scape lacking an extension (Fig. 4), while in *C. schulzi* sp. nov. the extension is well marked (Fig. 3). *Camponotus nitidescens* is the most similar to *C. schulzi* sp. nov., because both species have the mesosomal surface partly covered with weaker sculpture and especially the sides of mesosoma appear more or less shiny in both (Figs 15, 16). However, *C. nitidescens* has the base of the antennal scape without extension (Fig. 4) while in *C. schulzi* sp. nov. the extension is well marked (Fig. 3). Both species are also broadly separated geographically. *Camponotus nitidescens* has a narrow distribution range limited to the southern Ionian Islands, western Sterea Ellas, and Peloponnese. While *C. schulzi* sp. nov. was collected in western Turkey (Fig. 26). Species of the *C. piceus* complex of the *Camponotus lateralis* group at first glance can appear similar to *C. schulzi* sp. nov. but they differ in less-sculptured mesosoma and gaster. Especially their gaster is shinier and not as regularly reticulate or granulate as in *C. schulzi* sp. nov.

**Figures 17–22.**
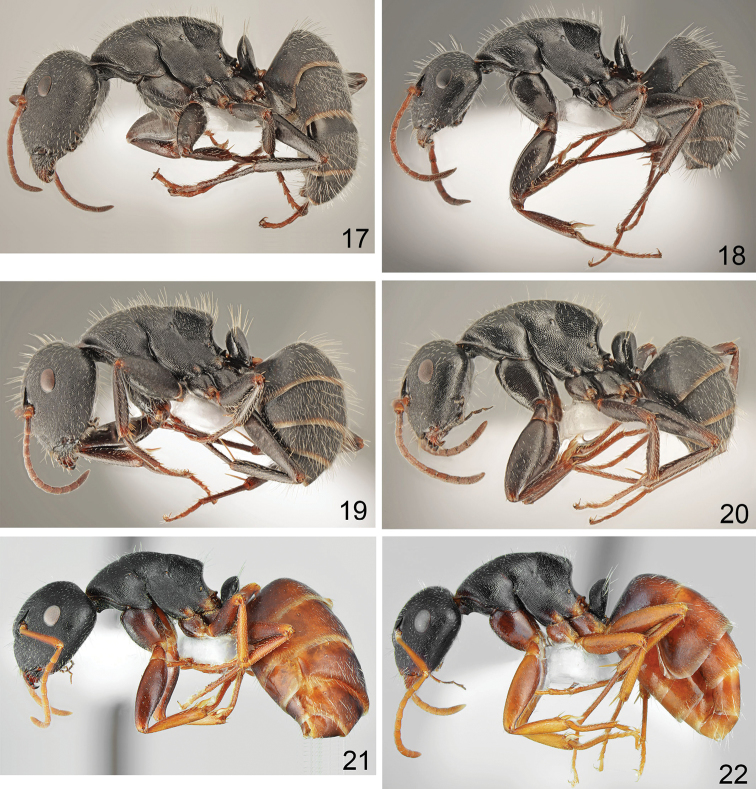
Workers in lateral view **17, 19, 21** major **18, 20, 22** minor: **17, 18***Camponotus aegaeus* Emery **19, 20***C. libanicus* André **21, 22***C. aktaci* Karaman.

## Discussion

*Camponotus schulzi* sp. nov. is a member of the subgenus Myrmentoma. Currently, there are 24 species and one subspecies of this subgenus known from the eastern part of the Mediterranean. Emery (1925) and Radchenko (1997) divided members of this subgenus into three groups: *Camponotus lateralis* group, *Camponotus fallax* group, and *Camponotus kiesenwetteri* group.

The *Camponotus lateralis* group is the most speciose and represented by 12 species: *C. anatolicus* Karaman & Aktaç, 2013, *C. atricolor* (Nylander, 1849), *C. candiotes* Emery, 1894, *C. dalmaticus* (Nylander, 1849), *C. ebneri* Finzi, 1930, *C. heidrunvogtae* Seifert, 2019, *C. hirtus* Karaman & Aktaç, 2013, *C. honaziensis* Karaman & Aktaç, 2013, *C. lateralis* (Olivier, 1792), *C. piceus* (Leach, 1825), *C. rebeccae* Forel, 1913, and *C. staryi* Pisarski, 1971. In the most recent revision of the group (Seifert 2019) its members were characterized by small body size, rectangular or trapezoid propodeum in dorsal view, propodeal dorsum clearly delimited laterally by strong longitudinal edges, discontinuous dorsal profile of mesosoma, which is always depressed between mesonotum and propodeum, straight to convex dorsal area of propodeum which forms a distinct angle with the caudal declivity, shiny gaster, and short and sparse pubescence on gaster. Species of this group occur in Europe, Asia Minor, and the Caucasus.

**Figures 23–30.**
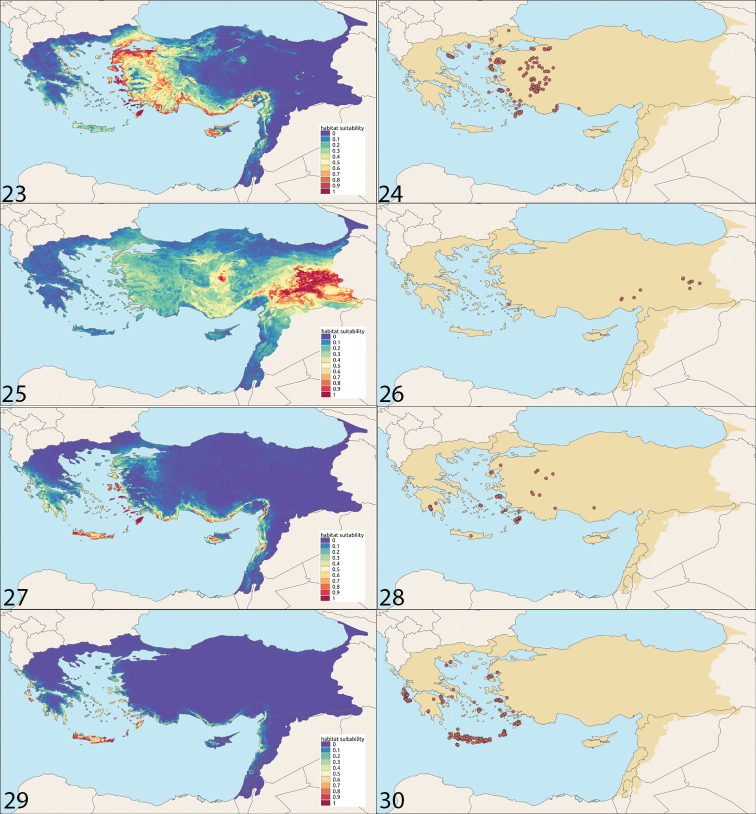
Habitat suitability and distribution **23, 24***Camponotus aegaeus***25, 26***Camponotus aktaci***27, 28***Camponotus boghossiani***29, 30***Camponotus kiesenwettri*.

The *Camponotus fallax* group contains six species and one subspecies – *C. abrahami* Forel, 1913, *C. fallax* (Nylander, 1856), *C. gestroi* Emery, 1878, *C. gestroi creticus* Forel, 1886, *C. kurdistanicus* Emery, 1898, *C. tergestinus* Müller, 1921, and *C. vogti* Forel, 1906. The group is characterized by a small to moderate body size, regularly arched mesosoma sometimes with shallow concavity between mesonotum and propodeum, straight to angular dorsal surface of propodeum, shiny surface of mesosoma and gaster, and short and never dense pubescence hairs on gaster.

The *Camponotus kiesenwetteri* group as defined here comprises seven species and can be divided into two groups. The first one consists of species lacking a metanotal groove and includes *C. aegaeus*, *C. libanicus*, and *C. aktaci*. The second group is created by taxa with shallow but distinct metanotal groove: *C. boghossiani*, *C. kiesenwetteri*, *C. nitidescens*, and *C. schulzi*. Most of the members of the *kiesenwetteri* group have an exclusively Aegean distribution. However, based on the distribution patterns of *C. libanicus*, and *C. aktaci* more records of members of this group are expected from the Near East. In fact, all species but *C. kiesenwetteri* showed large areas of suitable habitats in the east portion of the Aegean region.

**Figures 31–34.**
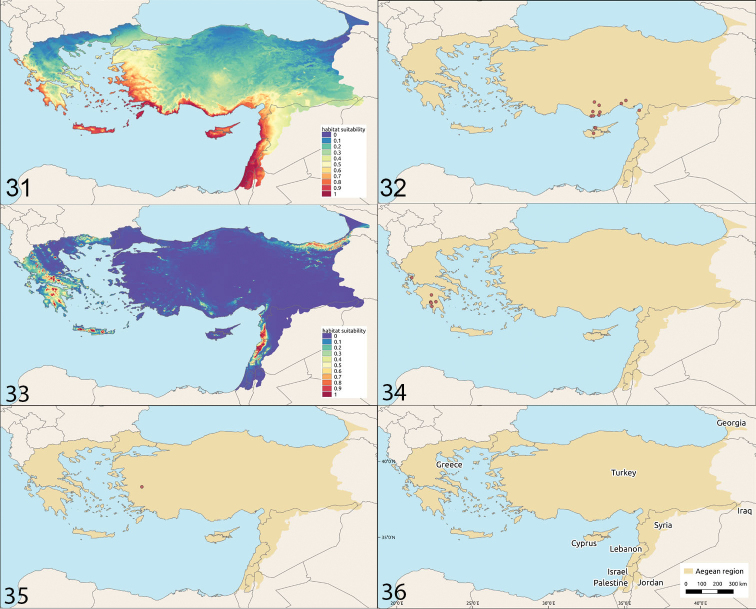
Habitat suitability and distribution **31, 32***Camponotus libanicus***33, 34***Camponotus nitidescens***35** distribution of *Camponotus schulzi***36** the Aegean with adjacent regions.

## Supplementary Material

XML Treatment for Camponotus kiesenwetteri

XML Treatment for Camponotus aegaeus

XML Treatment for Camponotus aktaci

XML Treatment for Camponotus boghossiani

XML Treatment for Camponotus kiesenwetteri

XML Treatment for Camponotus libanicus

XML Treatment for Camponotus nitidescens

XML Treatment for Camponotus schulzi
